# Fatal myositis and spontaneous haematoma induced by combined immune checkpoint inhibitor treatment in a patient with pancreatic adenocarcinoma

**DOI:** 10.1186/s12885-019-6372-z

**Published:** 2019-12-05

**Authors:** Yuan Liu, Zhi Liu, Xuejun Zeng, Chunmei Bai, Lin Chen, Songbai Lin, Xinlun Tian

**Affiliations:** 1 Department of Internal Medicine, Peking Union Medical College Hospital, Chinese Academy of Medical Sciences & Peking Union Medical College Hospital, Beijing, China; 2 Department of Neurology, Peking Union Medical College Hospital, Chinese Academy of Medical Sciences & Peking Union Medical College Hospital, Beijing, China; 3 Department of General Medicine, Peking Union Medical College Hospital, Chinese Academy of Medical Sciences & Peking Union Medical College Hospital, Beijing, China; 4 Department of Oncology, Peking Union Medical College Hospital, Chinese Academy of Medical Sciences & Peking Union Medical College Hospital, Beijing, China; 5 Department of international Medical Service, Peking Union Medical College Hospital, Chinese Academy of Medical Sciences & Peking Union Medical College Hospital, Beijing, China; 6 Department of Respiratory and Critical Care Medicine, Peking Union Medical College Hospital, Chinese Academy of Medical Sciences & Peking Union Medical College Hospital, #1 Shuaifuyuan, Wangfujing, Beijing, 100730 China

**Keywords:** Immune checkpoint inhibitors, Ipilimumab, Nivolumab, Myositis, Haematoma, Immune-related adverse event

## Abstract

**Background:**

Immune checkpoint inhibitors (ICIs) have achieved unprecedented success in cancer treatment over the past decade. The application of ICIs hasled to the discovery of various types of immune-related adverse events (irAEs). Here, we report a case of fatal myositis and spontaneous haematoma following concurrent treatment of nivolumab and ipilimumab for pancreatic adenocarcinoma.

**Case presentation:**

A 71-year-old gentleman with pancreatic adenocarcinoma underwent the Whipple procedure in September 2014. The patient received 8 cycles of adjuvant chemotherapy with gemcitabineand achieved a complete responsein April 2015. Treatment with the PD-1 inhibitor nivolumab was started due to suspected tumour recurrence in November 2015. In August 2016, the CTLA-4 inhibitor ipilimumab was added to nivolumab for 2 cycles. Eight weeks after the last dose, the patient developed severe myositis complicated with spontaneous haematomain skeletalmuscle. Pathology of the skeletal muscle autopsy revealed lymphocytic infiltration. Intense immunosuppressive therapy, including high-dose corticosteroids and methotrexate, resulted in clinical success in the treatment of myositis. However, the patient died of cancer recurrence.

**Conclusion:**

Myositis due to immunotherapy can be a fatal adverse event of ICIs, which requires close monitoring and cautious management.

## Background

Immune checkpoint inhibitors (ICIs), such as cytotoxic T-lymphocyte antigen-4 (CTLA-4) inhibition, target programmed death-1 (PD-1) and its ligand PD-L1, have been intensively investigated and developed to treat various types of cancer over the past decade [1]. Despite the fact that immunotherapy with checkpoint blockade has shown remarkable and durable responses in various cancer types, the application of checkpoint inhibitors in pancreatic cancer has been disappointing thus far [2]. In October 2015, the first combination of different ICIs (the PD-1 antibody Opdivo and the CTLA-4 antibody Yervoy) was approved for advanced malignant melanoma. Combined ICIs treatment has greatly improved the clinical outcomes of these cancers [3, 4]. However, a spectrum of autoimmune side effects, including rash, colitis, pneumonitis and endocrinopathies, was noted during clinical trials and post-marketing surveillance. These effects were referred to as immune-related adverse effects (irAEs) [5–10]. The frequency and severity of irAEs are even greater inpatients undergoing combined ICIs therapy [11–16]. Here, we report a case of fatal myositis complicated with spontaneous haematoma of the skeletal muscle after administration of Opdivo and Yervoy, which we hope will remind physicians to monitor the side effects and use these medications more discreetly.

## Case report

The patient was a 71-year-old gentleman diagnosed with pancreatic adenocarcinoma who was otherwise healthy with no family and psychosocial history. His tumour was first indicated by jaundice and a suspicious mass in the caput pancreatic in June 2014 with elevated serum carbohydrate antigen 19–9 (CA199) level (160.3 U/mL). Therefore, he underwent the Whipple procedure in September 2014. Post-operative pathology showed multiple areas of moderately differentiated adenocarcinoma. BRAF mutation and a variant of BRCA2 of unknown significance were noted. PD-L1 was negative. The patient received 8 cycles of adjuvant chemotherapy with gemcitabine and achieved a complete response in April 2015. There were no visible lesions, and his CA199 level dropped to 24 U/mL. However, tumour recurrence was suspected in November 2015 due to newly found lymph nodes with increased activity in the left supra and infra clavicle areas, porta hepatic area and peritoneal area according to positron emission tomography (PET). Treatment with the PD-1 inhibitor Opdivo (1 mg/kg) was started at a private hospital in foreign country together with capecitabine plus oxaliplatin (XELOX), Avastin and Zelboraf for 8 cycles with good tolerance, and then the patient was switched to Onyyvide, Xeloda, Avastin along with Opdivo. In August 2016, the CTLA-4 inhibitor Yervoy (3 mg/kg) was added to Opdivo for 2 cycles. Subsequent PET/CT showed decreased activity and size in lymph nodes and no evidence for recurrence.

Eight weeks after the last dose of Opdivo and Yervoy, the patient developed myalgia and myasthenia and presented to our emergency room on October 31, 2016 with a high level of creatine kinase (CK) (6235 U/L). Isozyme electrophoresis demonstrated that 95.4% of CK was creatine kinase-MM (CK-MM). However, his CK level continued to increase, and his symptoms worsened. An elevated erythrocyte sedimentation rate (33 mm/h) and hypersensitive C-reactive protein (3.88 mg/L) levels were detected together with positive serum anti-Ro-52 antibody. Electromyogram (EMG) indicated active myogenic damage (Additional file 1). Furthermore, biopsy of the right quadriceps femoris muscle was performed and revealed necrosis of skeletal muscle fibres and inflammationin the interstitial area and small vessels (Additional file 2). The severity of myasthenia continued to progress. Moreover, his swallowing muscles and respiratory muscles were involved, resulting in dysphagia and respiratory failure. Because the patient was able to drink water and enteral nutrition even when his symptoms were most serious, he was not intubated. Then, the patient was treated with methylprednisolone at an initial dose of 500 mg/d for 3 days starting November 10, 2016, followed by 60 mg/d intravenously. His symptoms improved over several weeks, and CK declined to normal from the highest level of 11,408 U/L.

**Fig. 1:**
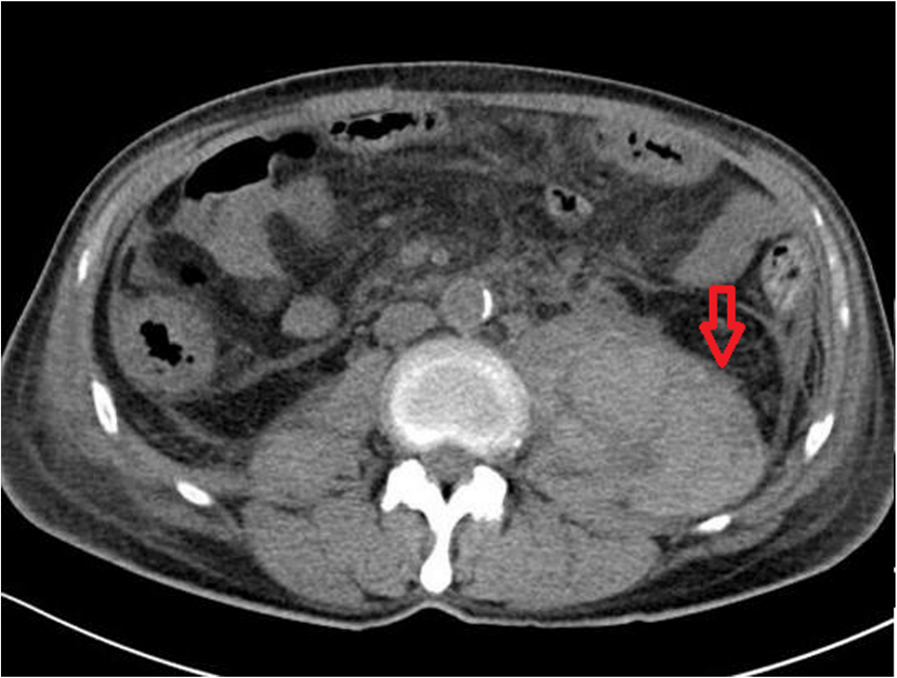
Radiographic imaging revealed a massive hematoma of the left psoas major muscle (arrow) (Enhanced abdominal CT was showed in Additional file 3)

However, 4 days after the initiation of glucocorticoid treatment, the patient developed acute and progressive low back pain radiating to the left lower limb. At the same time, a dramatic drop in haemoglobin from 120 g/L to 63 g/L within 48 h was noted. The patient’s platelet count was within the normal range throughout. Radiographic imaging revealed a massive haematoma of the left psoas major muscle (Fig. 1). The patient was on a preventive anticoagulant treatment of low-molecular-weight heparin (LMWH) (670 U/10 kg body weight every 12 h) because of his bed confinement status and low level of plasma albumin. No dysfunction of coagulation was detected, and no trauma had ever taken place. No petechia or bruising was noted after a thorough physical examination. Bleeding was quickly stopped after withdrawal of LMWH. A computed tomography angiography was performed, which found no leakage of contrast from the vessels. Additionally, the patient’s haemoglobin level remained stable. Interventional radiology was consulted, and vascular embolism was not recommended because the culpritvessel was difficult to determine. The patient was then discharged on December 9th with tapered oral glucocorticoids. Unfortunately, he died of pancreatic adenocarcinoma recurrence 2 months later with stable myositis.

**Fig. 2:**
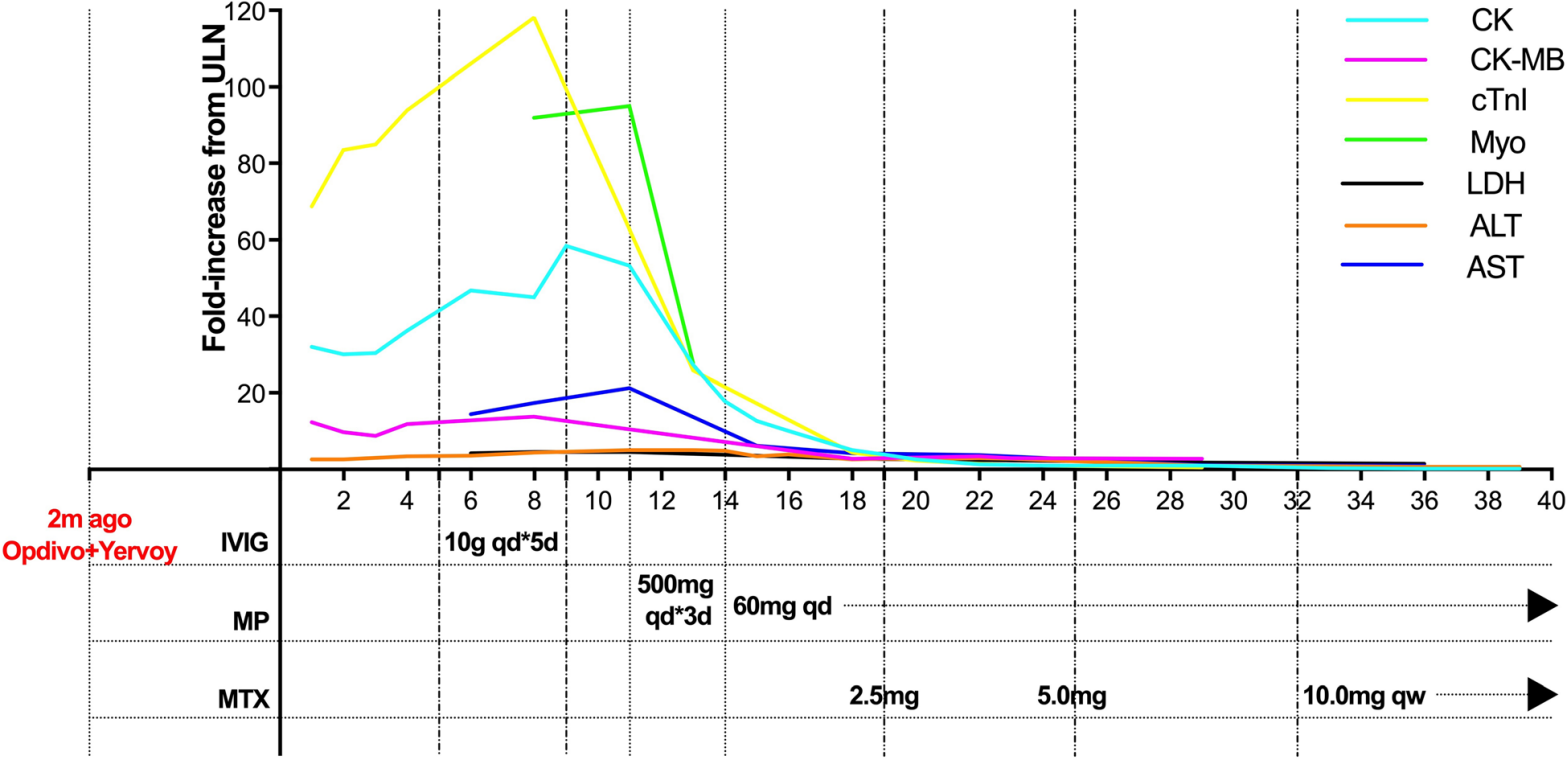
Timeline of interventions and laboratory results. ALT: glutamic-pyruvic transaminase, AST: cereal grass transaminase; CK: creatinekinase; CK-MB: creatine kinase-MB; cTnI: cardiac troponin I; IVIG: intravenous immunoglobulin; LDH:lactic acid dehydrogenase; LMWH: low molecular weight heparin; MP: methylprednisolone; MTX: methotrexate; Myo: myoglobin; ULN: upper limit of normal

Figure 2 shows the timeline of interventions and laboratory results after the patientwas diagnosed withpancreatic adenocarcinoma.

## Discussion

Myositis is rarely reported compared to other irAEs, such as dermatitis, endocrinopathies, colitis and pneumonitis. According to a previous study, the incidence of myositis in patients receiving ipilimumab plus nivolumab was 0.24% [5]. ICI-related myositis mimics primary dermatomyositis in clinical and laboratory features [17]. Most of the cases were manageable with glucocorticoids. Multi-disciplinary cooperation with rheumatologists is crucial. In our case, myositis developed after only 2 cycles of combined ICIs therapy. The progression of muscle damage was fulminant. The condition was nearly fatal. We suggest that clinicians remain vigilant for this rare irAE.

Here, we review 8 other reported cases of muscular haematoma in myositis since 1998 from the literature and summarize the clinical characteristics of muscular haematoma in myositis, including our case (Table 1). Seven out of 9 patients are above the age of 60. The median age is 64. The sex ratio is 1: 2 (male: female). This illustrates that elderly female patients are more likely to suffer from muscular haematoma. Muscles of the retroperitoneal area, including the iliopsoas muscle and psoas muscle, are more prone to spontaneous bleeding. The aetiology of the susceptibility of the iliopsoas to spontaneous haemorrhage is unclear. A proposed theory is that the iliopsoas is naturally predisposed to spontaneous intramuscular haemorrhage, as they are the strongest flexors in the body and are involved in numerous locomotive manoeuvres [25]. Seven out of 9 patients received anticoagulation treatment, both unfractionated heparin (UFH) and LMWH. There is no evidence to prove that any anticoagulant is safer than others regarding the risk of muscle bleeding. Moreover, those patients who received anticoagulant also underwent glucocorticoid pulse treatment, which may suggestmore severe myositis. This interesting coincidence makes corticosteroids truly suspicious of causing spontaneous haematoma. However, there is no literature proving a definite relationship between steroid and spontaneous haematoma. Except for our case, none of the 8 cases were diagnosed with malignancy.

**Table 1:** Clinical characteristics of muscle haematoma in myositis from the literature [18–24]

Case	Age (years old)	Sex	Underlying diseases	Bleeding site	Anticoagulant drugs	Coagulability	Intravenous methylprednisolone pulse
1[18]	50	Female	No	Left rectus abdomens	No	Normal	No
2[18]	11	Female	No	Right retroperitoneum	No	Normal	No
3[19]	80	Male	No	Left rectus sheath, oblique right thigh	UFH	APTT prolonged	Yes
4[20]	77	Female	No	Left iliopsoas iliac, retroperitoneum	UFH	APTT prolonged	Yes
5[21]	64	Female	No	Right retroperitoneum, left rectus sheath	Dalteparin	Normal	Yes
6[22]	65	Female	No	Iliopsoas both sides, thigh	UFH	APTT prolonged	Yes
7[23]	60	Male	No	Left trapezius	UFH	APTT prolonged	Yes
8[24]	60	Female	No	Left psoas	Enoxaparin	Normal	Yes
9(our case)	71	Male	Pancreatic adenocarcinoma	Left psoas major	Enoxaparin	Normal	Yes

To the best of our knowledge, this is the first case report of life-threatening myositis and spontaneous muscular haematoma associated with combined ICIs therapy since pancreatic adenocarcinoma is immune quiescent. To date, checkpoint inhibition therapy has failed to elicit efficacy in patients with pancreatic cancer [26–29]. Combination regimens comprising chemotherapy and ICIs have shown initial promise in clinical trials and in animal studies, but these results need to be verified [30–38]. We believe it was not rigorous to administer this combined treatment to pancreatic cancer patients. In addition to spontaneous haematomas, other severe complications of myositis, such as acute rhabdomyolysis, have also been reported with ipilimumab-nivolumab treatment as mere associations [17]. However, we cannot conclude that ICIs contribute to these severe complications. Nevertheless, our report emphasized the necessity of closely monitoring irAEs in patients treated with combination immunotherapy. Meanwhile, the potential danger of anticoagulation therapy in a patient treated with ICIs, especially in the elderly population, should be alerted. Thus, clarity the indication and strict clinical surveillance would be of value.

## Supplementary information

**Additional file 1:** Electromyogram result.

**Additional file 2:** Pathological image of biopsy of the right quadriceps femoris muscle.

**Additional file 3:** Figure of enchanced CT image of haematoma of the left psoas major muscle.

## Data Availability

All data generated or analyzed during this study are included in this published article.

## Abbreviations

CA199: Carbohydrate antigen 19–9
CK: Creatinekinase
CTLA-4: Cytotoxic T-lymphocyte antigen-4
EMG: Electromyogram
ICIs: Immune checkpoints inhibitors
irAEs: Immune-related adverse effects
LMWH: Low molecular weight heparin
MP: Methylprednisolone
PD-1: Programmed death-1
PET: Positron emission tomography
UFH: Unfractionated heparin
XELOX: Capecitabine plus oxaliplatin
