# Supplementary material for: Fatal myositis and spontaneous haematoma induced by combined immune checkpoint inhibitor treatment in a patient with pancreatic adenocarcinoma
Source: BMC Cancer. 2019 Dec 5;19:1193. doi: 10.1186/s12885-019-6372-z (PMC6896742; doi:10.1186/s12885-019-6372-z)
Supplement: Supplementary file 1 — Additional file 1: Electromyogram result. [file 12885_2019_6372_MOESM1_ESM.docx]

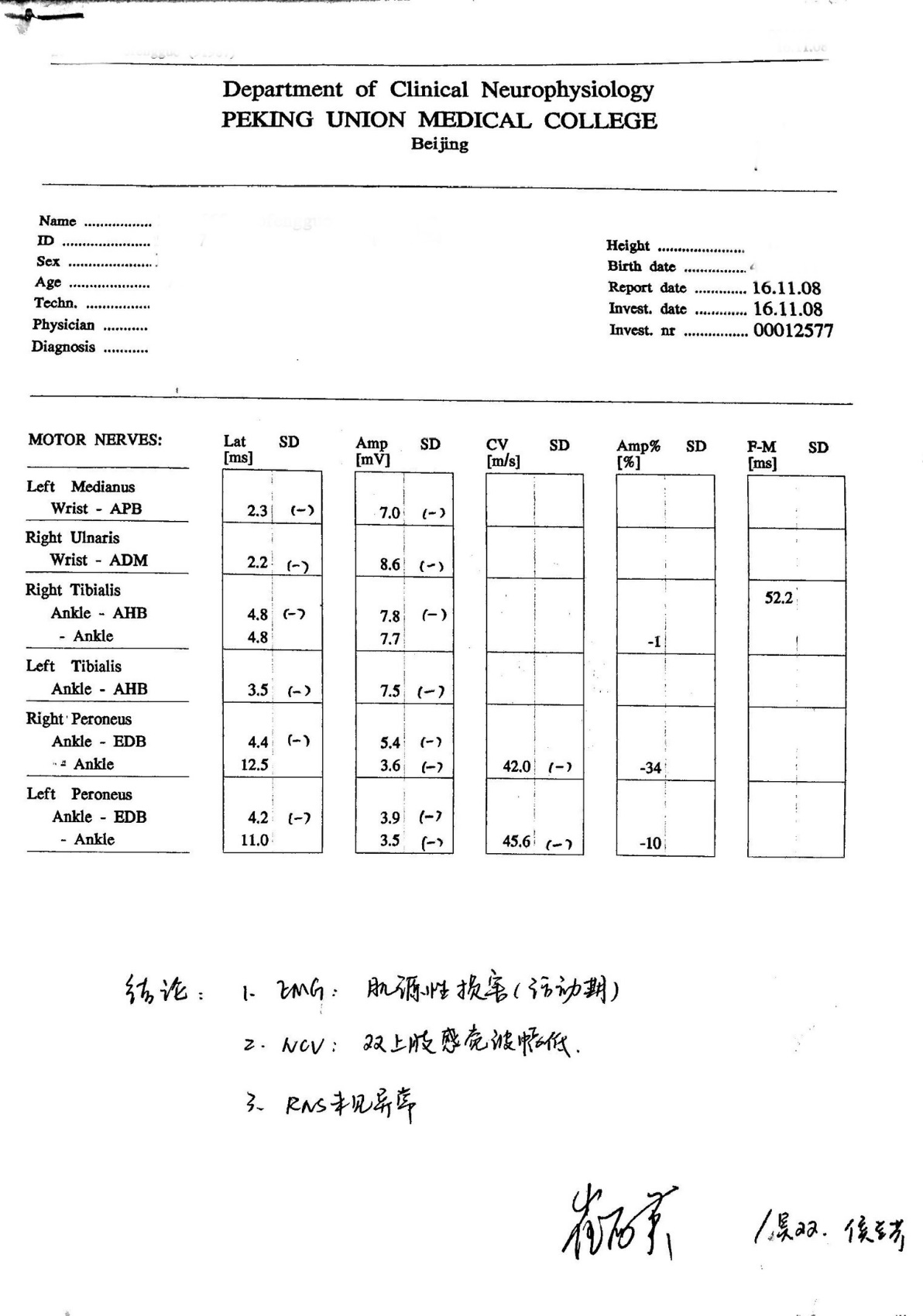


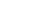


Conclusion:

1. EMG: Active myogenic damage.
2. NCV: Sensory nerve amplitude of both upper limbs was decreased.
3. RNS: no abnormality noted.
