# Supplementary material for: Fatal myositis and spontaneous haematoma induced by combined immune checkpoint inhibitor treatment in a patient with pancreatic adenocarcinoma
Source: BMC Cancer. 2019 Dec 5;19:1193. doi: 10.1186/s12885-019-6372-z (PMC6896742; doi:10.1186/s12885-019-6372-z)
Supplement: Supplementary file 2 — Additional file 2: Pathological image of biopsy of the right quadriceps femoris muscle. [file 12885_2019_6372_MOESM2_ESM.doc]

A Quadriceps femoris staining with hematoxyl
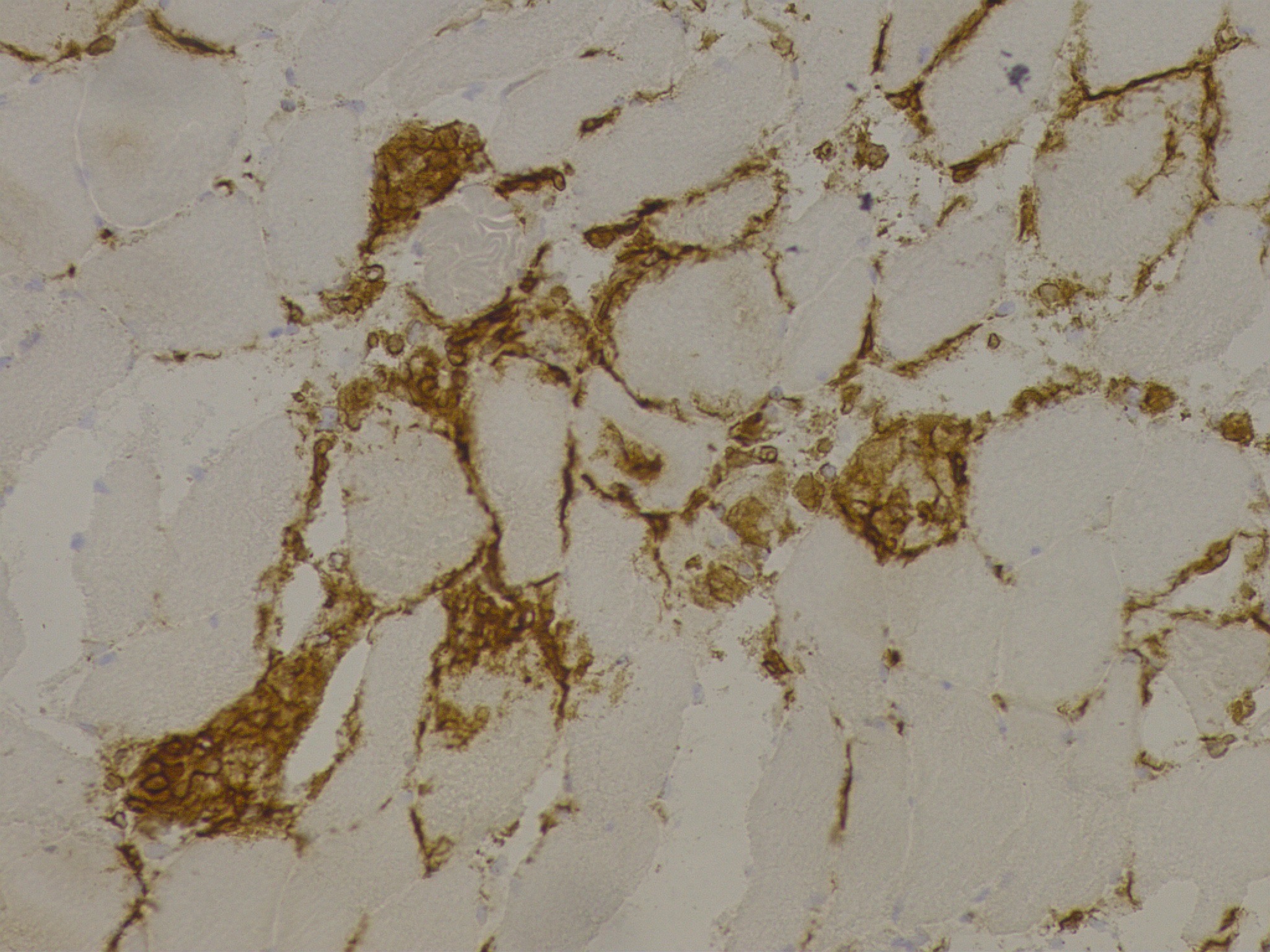

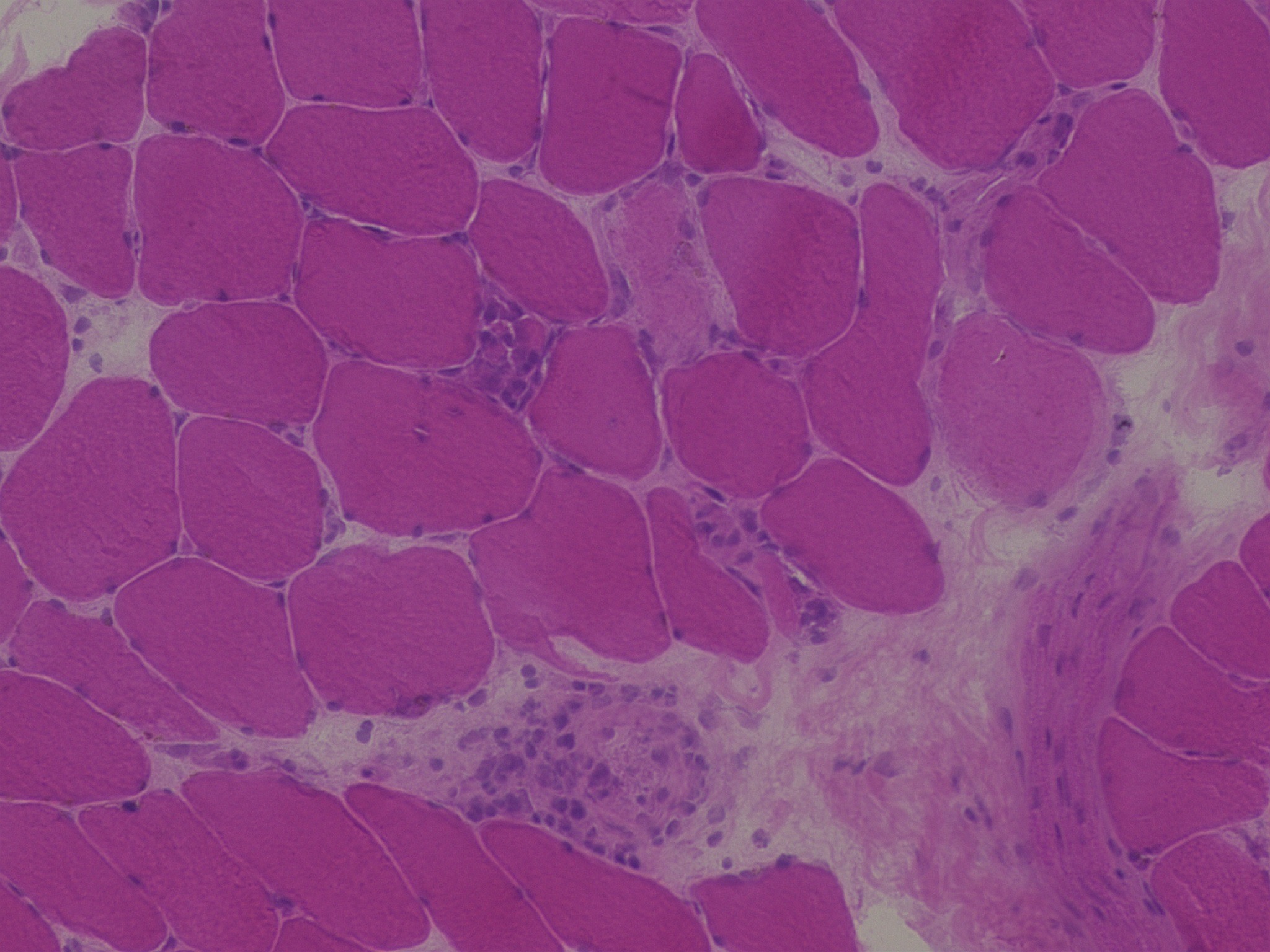
in and eosin

B Infiltrate with CD4+ T Cells

C Infiltrate wi
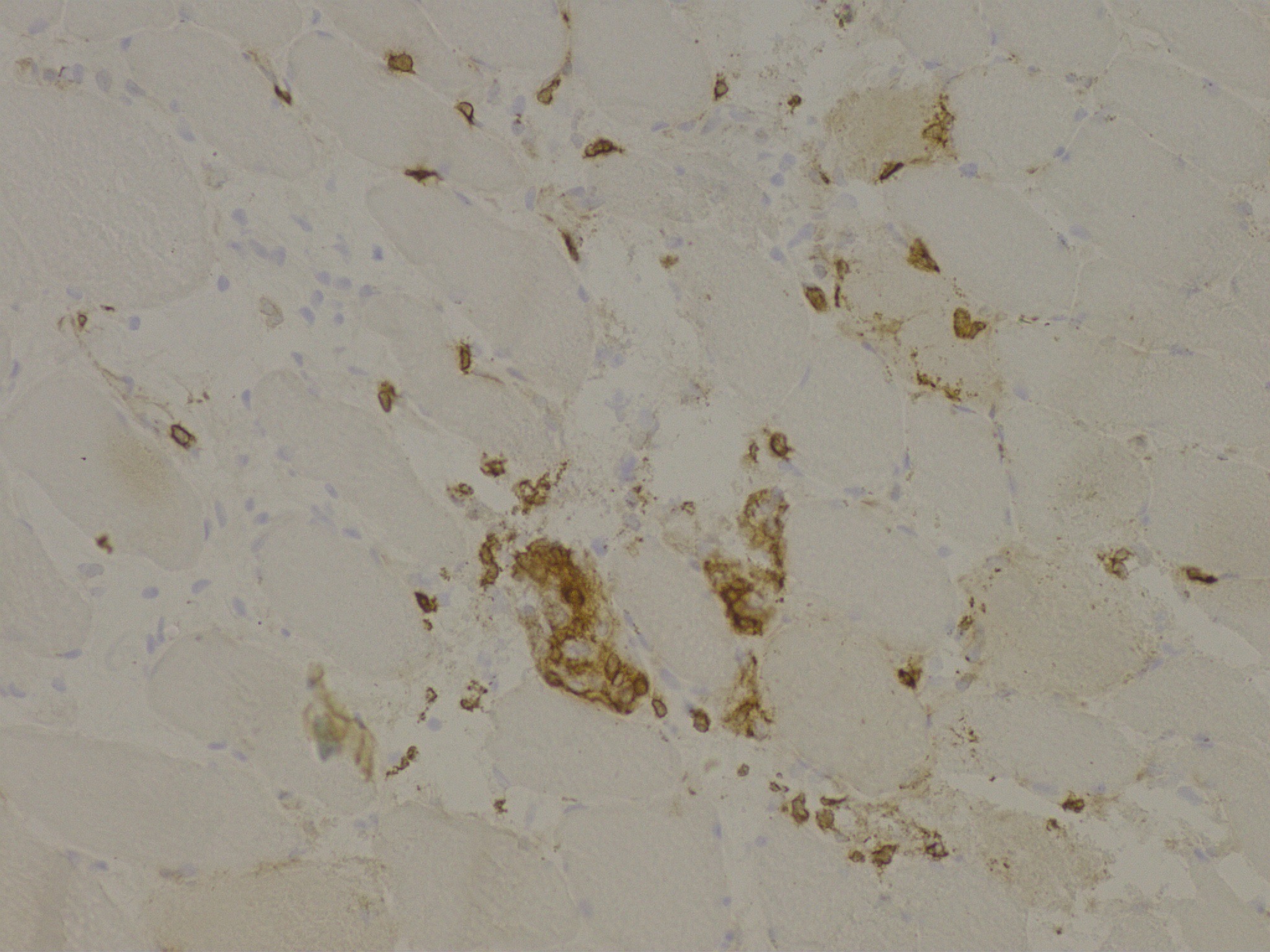

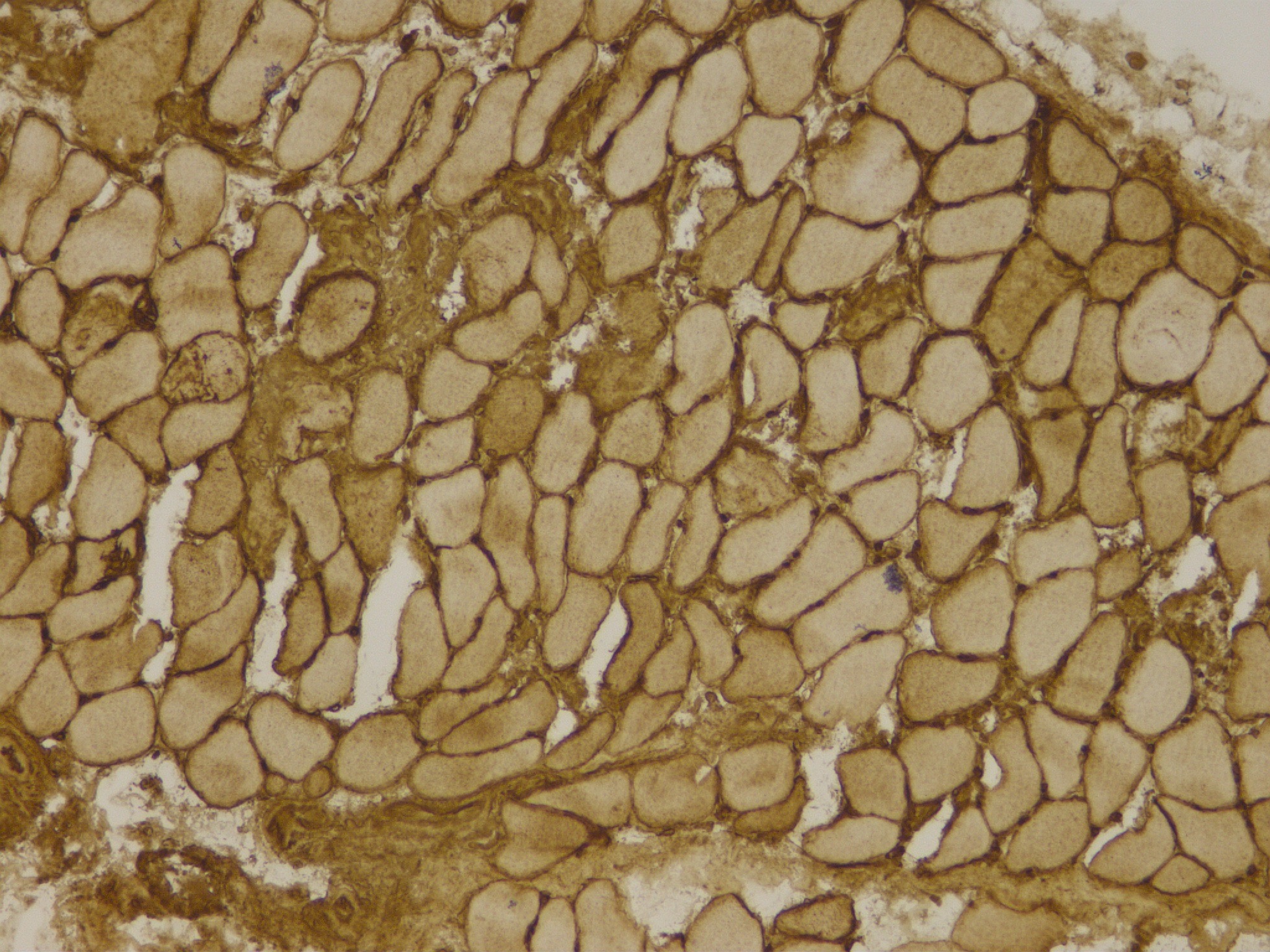
th CD8+ T cells

D Over-expression of MHC-1

Autopsy of skeletal muscle revealed lymphocytic infiltration. Many of the lymphocytic infiltrate were positive for CD8(B), fewer were positive for CD4(C). Expression of MHC-1 on sarcolemma was significantly elevated(D).
