# Supplementary figures and images for: Fatal myositis and spontaneous haematoma induced by combined immune checkpoint inhibitor treatment in a patient with pancreatic adenocarcinoma
Source: BMC Cancer. 2019 Dec 5;19:1193. doi: 10.1186/s12885-019-6372-z (PMC6896742; doi:10.1186/s12885-019-6372-z)

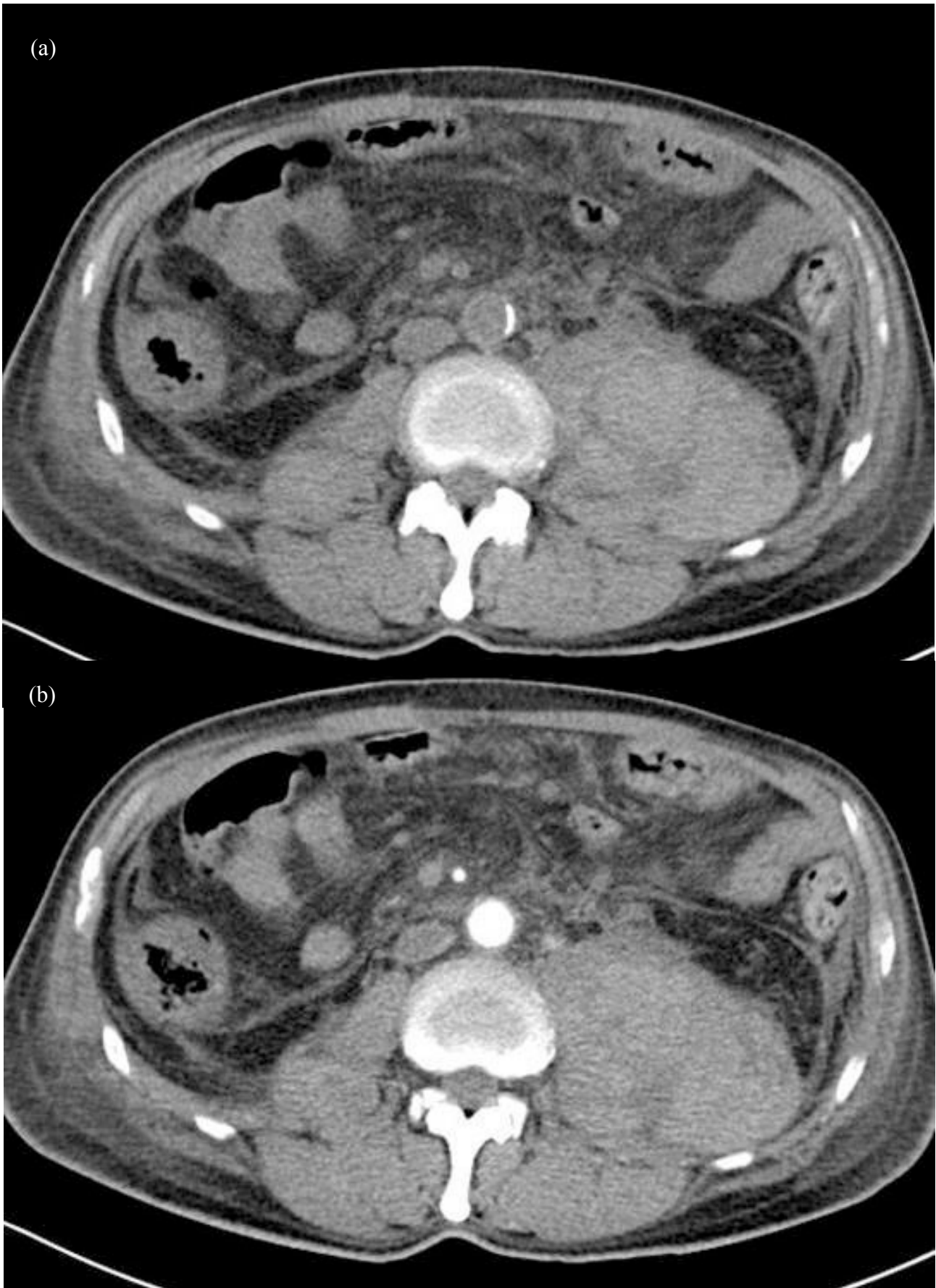

Figure 1 Abdominal CT(plain scan(a) and enhanced(b))showed hematoma of the left psoas major muscle.

Supplement: Supplementary file 3 — Additional file 3: Figure of enchanced CT image of haematoma of the left psoas major muscle. [file 12885_2019_6372_MOESM3_ESM.pdf]
